# Genetically predicted metabolites mediate the causal associations between autoimmune thyroiditis and immune cells

**DOI:** 10.3389/fendo.2024.1424957

**Published:** 2024-07-09

**Authors:** Yongzhao Chen, Bo Jiang, Cheng Qu, Chaoyu Jiang, Chen Zhang, Yanxue Wang, Fei Chen, Xitai Sun, Lei Su, Yuqian Luo

**Affiliations:** ^1^ Department of General Surgery, Nanjing Drum Tower Hospital, The Affiliated Hospital of Nanjing University of Chinese Medicine, Nanjing, China; ^2^ Department of General Surgery, Nanjing Drum Tower Hospital, The Affiliated Hospital of Nanjing University Medical School, Nanjing, China; ^3^ General Surgery Center, Department of Thyroid Surgery, Zhujiang Hospital, Southern Medical University, Guangzhou, China; ^4^ Division of Pancreas and Metabolism Surgery, Department of General Surgery, Nanjing Drum Tower Hospital, The Affiliated Hospital of Nanjing University of Chinese Medicine, Nanjing, China; ^5^ Clinical Medicine Research Center, Nanjing Drum Tower Hospital, The Affiliated Hospital of Nanjing University Medical School, Nanjing, China

**Keywords:** autoimmune thyroiditis, immune cells, metabolites, Mendelian randomization, mediation effects

## Abstract

**Introduction:**

We aimed to comprehensively investigate the causal relationship between 731 immune cell traits and autoimmune thyroiditis (AIT) and to identify and quantify the role of 1400 metabolic traits as potential mediators in between.

**Methods:**

Using summary-level data from genome-wide association studies (GWAS) we performed a two-sample bidirectional Mendelian randomization (MR) analysis of genetically predicted AIT and 731 immune cell traits. Furthermore, we used a two-step MR analysis to quantify the proportion of the total effects (that the immune cells exerted on the risk of AIT) mediated by potential metabolites.

**Results:**

We identified 24 immune cell traits (with odds ratio (OR) ranging from 1.3166 6 to 0.6323) and 10 metabolic traits (with OR ranging from 1.7954 to 0.6158) to be causally associated with AIT, respectively. Five immune cell traits (including CD38 on IgD+ CD24-, CD28 on CD28+ CD45RA+ CD8br, HLA DR+ CD4+ AC, TD CD4+ %CD4+, and CD8 on EM CD8br) were found to be associated with the risk of AIT, which were partially mediated by metabolites (including glycolithocholate sulfate, 5alpha-androstan-3alpha,17beta-diol disulfate, arachidonoylcholine, X-15486, and kynurenine). The proportion of genetically predicted AIT mediated by the identified metabolites could range from 5.58% to 17.7%.

**Discussion:**

Our study identified causal associations between AIT and immune cells which were partially mediated by metabolites, thus providing guidance for future clinical and basic research.

## 1 Introduction

Autoimmune thyroiditis (AIT), also known as Hashimoto’s thyroiditis (HT) or chronic lymphocytic thyroiditis, is an autoimmune disorder affecting the thyroid gland (1). In AIT, immune cells lead to the death of the thyroid’s hormone-producing cells, which could result in a decline in hormone production (i.e. hypothyroidism) (1). The primary treatment is thyroid hormone replacement. AIT affects about 5% of Caucasians at some point in their lives (1). It is the most common cause of hypothyroidism in iodine-sufficient areas of the world. It typically begins between the ages of 30 and 50 and is much more common in women than men. Rates of the disease appear to be increasing (1). It is generally believed that HT manifests through a combination of genetic susceptibility and environmental risk factors (2), nevertheless, the specific risk factors and detailed pathogenesis of AIT remained inconclusive.

AIT is a classic example of a T-cell-mediated disease in which thyroid cells is destroyed by an inflammatory infiltrate consisting of CD4+ and CD8+ T cells, CD19+ B cells, macrophages, and plasma cells (3). In terms of thyroid cell injury, cytokines derived from the inflammatory infiltrate play a key role, including their ability to stimulate the thyroid cells themselves to release pro-inflammatory mediators, thus amplifying and perpetuating the autoimmune response (2). CD4+ T cells can differentiate into different subtypes such as T helper cell 1 (Th1), Th2, Th17, and the regulatory T cells (Treg). Th1 cells activate killing lymphocytes (including CD8+T cells, macrophages, etc.) which are primarily responsible for the destruction of thyroid cells (3, 4). In AIT tissues, CD8+Tcytotoxic cells attack self-thyroid cells, causing cell death and tissue damage (3, 4). The functional imbalance between pro-inflammatory Th17 and immunosuppressive Treg is involved in the pathogenesis of various autoimmune diseases including AIT (2, 5). Cytokine productions of Th1 and Th2 dictate the activation of B cells to secrete antibodies (6). The majority of thyroid autoantibodies (i.e. TGAb and TPOAb) detected in the serum of HT patients are IgG secreted by activated B cells. HT can be classified into IgG4 and non-IgG4 subtypes based on the subtypes of thyroid autoantibodies (7). Patients with IgG4 HT are more likely to experience early onset hypothyroidism and thyroid atrophy (8). And it is reported that thyroid autoantibodies, particularly TGAb, are associated with recurrent miscarriage and could be an expression of a more general maternal immune system abnormality (9, 10). The close relationship between AIT and immune cells has been shown by extensive by observational studies. However, the causal associations between a specific immune cell trait and AIT remained somehow elusive, possibly due to limited sample sizes, flaws in study design, and confounding factors beyond the scope of the existing studies.

Metabolites are the end products of biochemical processes. Metabolites can induce permanent alterations in cellular organization and genetic architecture through the regulation of the epigenome, a phenomenon known as metabolic epigenetics (11). It has been proposed that aberrant energy metabolism disrupts immune tolerance, which may ultimately lead to autoimmune responses. The incidence of autoimmune might be attributed to the aberrations of metabolites in immune cells (12, 13). Moreover, studies have shown that immune cells are very sensitive to metabolites in the body and can rapidly adapt to the microenvironment of hypoglycemia and high lactate, thus participating in the maintenance of immune tolerance to self and in the control of metabolic homeostasis (14). Previous epidemiology studies have proved the crucial role of metabolites that participate in the reprogramming of various autoimmune diseases (15–17). Changes in serum metabolites have been reported in AIT. Jiang et al. reported that phospholipids influenced the pathogenesis of Hashimoto’s thyroiditis, and fatty acid degradation and lysine degradation pathways had an impact on different clinical stages of HT (18). Krupa et al. reported that the serum kynurenine metabolite profile was dysregulated in young women with AIT and could serve as a new predictor of AIT risk (19). Song et al. reported that patients with AIT had higher serum L-arginine, L-ornithine, lysine and agmatine levels, and lower putrescine, 1,3-diaminopropane, spermine, N-acetylputrescine levels; and N-acetylspermidine might be a risk factor for HT progression to overt hypothyroidism (20). The existing studies mainly focused on the development of novel diagnosis metabolic markers, while the causal relationship between specific metabolites and AIT and their potential mediation effects on the pathways from immune cells to AIT remained unclear.

Mendelian randomization (MR) is a potential causal inference method that uses genetic variation as an instrumental variable to obtain the effect of exposure factors on outcomes from observational data (21). MR can reduce the effects of non-measurement errors or confounding factors while avoiding reverse causality through Mendelian inheritance laws (21). In the present study, we aimed to determine the specific immune cell signature that was causally associated with AIT and to assess the extent to which a specific metabolic trait could mediate the effect of the immune cell on AIT.

## 2 Methods

### 2.1 Study design

**Figure 1:**
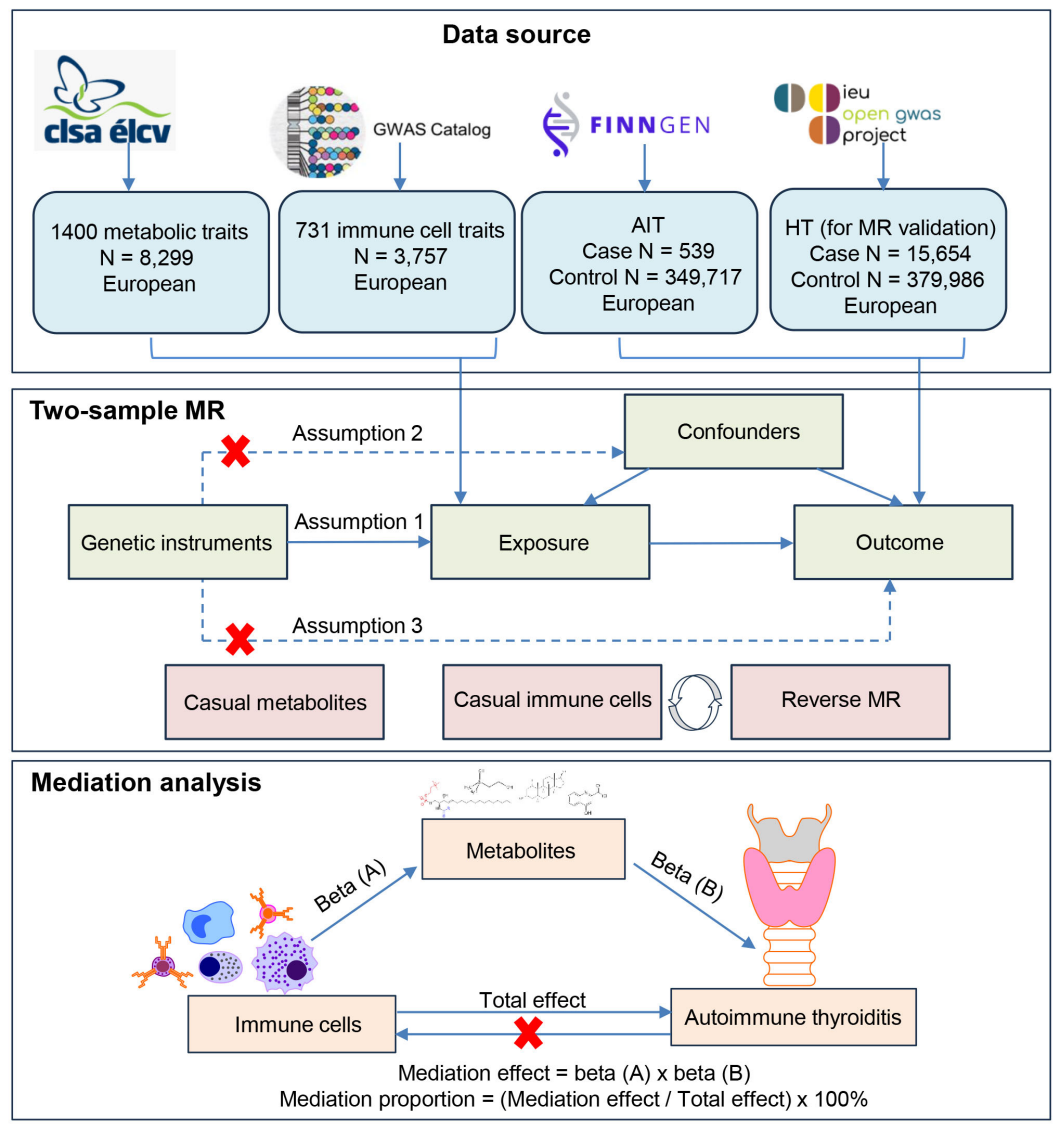
Flow chart illustrating the study design. First, we obtained published GWAS summary datasets that included traits including metabolites, immune cells, and AIT (also known as HT). Second, two-sample MR analyses were used to evaluate relationships among metabolites, immune cells, and AIT. Additionally, a reverse MR analysis was conducted between immune cells and AIT to enhance the robustness of the outcomes. A HT dataset was also used for MR validation. Our approach was firmly rooted in adhering to the fundamental tenets of MR analysis. Finally, mediation analysis was used to determine the mediation effect of metabolites on the relationship between immune cells and AIT.

**Figure 1** shows a schematic summary of the analysis. First, we obtained published GWAS summary datasets that included traits including metabolites, immune cells, and AIT (also known as HT). Second, two-sample MR analyses were used to evaluate the relationship among metabolites, immune cells, and AIT. Additionally, to reinforce the validity of our findings, a reverse MR analysis was conducted, leveraging significant results from the initial MR analysis between immune cells and AIT to enhance the robustness of the outcomes. Our approach was firmly rooted in adhering to the fundamental tenets of MR analysis. We ensured a robust association existing between genetic variants and the exposure (assumption 1); we ascertained that these genetic variants are not associated with potential confounding factors (assumption 2); and we confirmed that the impact of genetic variants on the outcome is mediated exclusively through the exposure, without the influence of alternative biological pathways (assumption 3). Finally, mediation analysis was used to determine the mediation effect of metabolites on the relationship between immune cells and AIT. This study is conducted and reported following the Strengthening the Reporting of Observational Studies in Epidemiology Using Mendelian Randomization guidelines (STROBE-MR) (22).

### 2.2 Genome-wide association study data sources

The datasets used in our analysis were publicly available and were approved by the institutional review committee in the respective studies. Therefore, no further sanctions were needed.

#### 2.2.1 GWAS data for AIT and HT

Data on AIT were drawn from the GWAS summary data sources on the FinnGen consortium (https://www.finngen.fi/en). AIT GWAS summary statistics consist of 350,256 individuals (539 AIT cases and 349,717 controls), which are available at https://r10.risteys.finregistry.fi/endpoints/E4_THYROIDITAUTOIM. Individuals with ICD codes [ICD-10 E06.3] “Autoimmune thyroiditis” including Hashimoto thyroiditis, hashitoxicosis (transient), lymphadenoid goiter, lymphocytic thyroiditis, struma lymphomatosa were defined as AIT cases (https://icd.who.int/browse10/2016/en#/E06.3).

Data on HT for MR validation were drawn from IEU Open GWAS project (dataset: ebi-a-GCST90018855). HT GWAS summary statistics consist of 395,640 European individuals (15,654 HT cases and 379,986 controls), which can be downloaded from GWAS catalog (https://www.ebi.ac.uk/gwas/) under the accession number GCST90018855. A total of 24,146,037 SNPs were included in this dataset (23).

#### 2.2.2 GWAS data for immune cells

GWAS summary statistics for each immune cell traits are publicly available from the GWAS Catalog (accession numbers from GCST0001391 to GCST0002121) (24). A total of 731 immune signatures including 7 panels of B cells, dendritic cells (DC), mature stages of T cells, monocytes, myeloid cells, TBNK (T cells, B cells, natural killer cells), and Treg. The original GWAS on immune cells was performed using data from 3,757 European individuals and there were no overlapping cohorts. Approximately 22 million SNPs genotyped with high-density arrays were imputed with Sardinian sequence-based reference panel and associations were tested after adjusting for covariates (i.e., sex, age and age^2^). This immune cell dataset detected 122 significant independent association signals for 459 cell traits and 70 loci (53 of them novel); 53 signals at 36 loci overlapped with previously reported disease-associated signals, predominantly for autoimmune disorders, highlighting intermediated phenotypes in pathogenesis (25).

#### 2.2.3 GWAS data for metabolites

GWAS summary statistics for each metabolic trait are publicly available from the GWAS Catalog (accession numbers from GCST90199621 to GCST90201020) (26). A total of 1400 metabolic signatures included 1091 blood metabolites and 309 metabolite ratios in 8,299 individuals of European descent from the Canadian Longitudinal Study on Aging (CLSA) cohort (26). This is the currently most comprehensive analysis of plasma metabolome metabolites implicated in human diseases which included approximately 15.4 million SNPs (26). Of the 1,091 plasma metabolites tested, 850 had known identities across eight super pathways (i.e., lipid, amino acid, xenobiotics, nucleotide, cofactor and vitamins, carbohydrate, peptide, and energy). The remaining 241 were categorized as unknown or partially characterized molecules (26). Metabolite ratios included the ratio of substrates to products of enzymatic reactions, and the metabolite level ratios for metabolite pairs sharing an enzyme or transporter (26).

### 2.3 Selection of instrumental variables

To estimate causal effects using genetic variation, three basic assumptions of IVs must be satisfied: (1) genetic variation is directly associated with exposure; (2) genetic variation is not associated with possible confounders between exposure and outcome; and (3) genetic variation does not affect outcome through pathways other than exposure. In accordance with recent research (24), the selection criteria for identifying IVs were as follows: a) SNPs linked to each genus with locus-wide significance (P < 1 × 10^−5^) were considered as potential IVs; b) Data from the European samples within the 1000 Genomes Project served as the reference panel for calculating linkage disequilibrium (LD) among the SNPs (27). SNPs with an R^2^ value of less than 0.001 (using a clumping window size of 10,000 kb) were further analyzed, and only those SNPs exhibiting the most significant P-values were retained for subsequent analysis; c) The F statistic was calculated by the variance explained by SNPs for each exposure, i.e. [(N – K – 1)/K]/[R^2^/(1 – R^2^)], where K is the number of genetic variants, N is the sample size. SNPs with an F statistic <10 should be excluded (28).

### 2.4 Statistical analysis

#### 2.4.1 Primary analysis: two-sample bidirectional MR analysis

We conducted a two-sample bidirectional MR to evaluate the mutual causality between 731 immune cell traits and AIT (**Figure 1**), which was designated as the total effect. Inverse variance weighting (IVW) uses meta-analysis to combine the Wald ratios of causal effects for each single nucleotide polymorphism (SNP) (29). Then, MR-Egger (30), weighted-median (31), simple mode, and weighted mode methods (32) were used as a complement to IVW. We initiated by harmonizing SNPs with identical alleles from the data source, followed by conducting a two-sample MR analysis. The IVW method integrates Wald estimates from each SNP through meta-analysis, providing a comprehensive estimation of the influence of immune cells on AIT. Its advantage lies in simultaneously considering the effects of multiple genotypes on the study factor, thereby enhancing the accuracy of causal inference. The IVW result remains unbiased in the absence of horizontal pleiotropy (33). We utilized odds ratios (ORs) of the exponential β for categorical outcomes along with corresponding CIs to estimate effect sizes of causality. A significance threshold of P < 0.05 was applied (or otherwise indicated). All the analyses were done by R package named “TwoSampleMR” within the environment of R 4.3.2 (available at https://cran.r-project.org/bin/windows/base/). To ensure the robustness and sensitivity of our findings, we also performed additional analyses, including MR-Egger, weighted median, simple mode, and weighted mode. The MR-Egger method is a commonly employed randomization pattern in Mendelian randomization, assessing the impact of a factor on disease through a linear regression model. Egger regression is utilized to estimate bias and correct results, enhancing the accuracy of causal estimates (34). The weighted median method is primarily applied to address biased samples, effectively mitigating sample bias and improving the reliability and accuracy of randomized experiments (31). Both the simple mode and weighted mode are frequently implemented randomization patterns that eliminate interfering factors in experimental results by random grouping (35). The reverse MR analysis procedure was similar to that used for the MR analysis with AIT as the exposure while immune cells as the outcome. The HT dataset was used for MR validation, and the procedure was the same as the above described. Missing values were not inferred.

#### 2.4.2 Mediation analysis

Mediation analysis aims to evaluate the pathway from exposure to outcome through a mediator, which helps explore the potential mechanisms by which exposure affects outcome (24). We performed a mediation analysis using a two-step MR design to explore whether 1400 metabolic traits mediate the causal pathway from immune cells to AIT (**Figure 1**). The total effect of immune cells on AIT can be decomposed into an indirect effect and a direct effect (36). First, the causal relationship between metabolites and immune cells was evaluated using two-sample MR methods to obtain beta (A). Second, two-sample MR was used to screen metabolites that still had a causal relationship with AIT after correction for immune cells to obtain beta (B) and ensure that the mediating effects on outcomes are independent of exposure (36). The mediation effect was thus calculated using a two-step MR: mediation effect = beta (A) × beta (B). The total effect of the immune cells on AIT was obtained in the previous two-sample MR, and direct effect = (total effect − mediation effect). The mediation proportion used the following formula: mediation proportion = (mediation effect/total effect) × 100%. The 95% confidence intervals (CI) for the mediation effects and proportions mediated were estimated using the delta method (36). Based on the results, we categorized the mediators into different levels of evidence. When only a triangular relationship existed, the metabolites were considered to have potential mediation effects in the pathway from immune cells to AIT (**Figure 1**).

### 2.5 Sensitivity analysis

Heterogeneity was assessed using Cochrane’s Q test calculated in the IVW methods while potential pleiotropy was evaluated and corrected using the MR-Egger intercept test. Cochran’s Q test is employed as a method to evaluate heterogeneity among different IVs in a study (37). The P-value derived from Cochran’s Q test is crucial in determining the presence or absence of significant heterogeneity. If the P-value is less than the pre-defined significance level (usually 0.05), it is indicative of significant heterogeneity among the IVs (37).To assess the effect of horizontal pleiotropy, a common method was used (i.e., MR-Egger), which implies the presence of horizontal multiplicity if its intercept term is significant (30). Furthermore, a powerful method, the MR pleiotropy residual sum and outlier (MR-PRESSO) method was utilized to exclude possible horizontal pleiotropic outliers that could substantially affect the estimation results in the MR-PRESSO package (38). In addition, scatter plots and funnel plots were generated. Scatter plots showed that the results were not affected by outliers. Funnel plots demonstrated the robustness of the correlation and no heterogeneity. Finally, the “leave-one-out” method was employed to evaluate the causal genetic impact of potential outlier SNPs and to ascertain whether the exclusion of these SNPs influenced the MR estimates.

### 2.6 Metabolic enrichment analysis

For identified known plasma metabolites (P < 0.05 at least in IVW method), we used MetaboAnalyst 6.0 (https://www.metaboanalyst.ca/) to conduct metabolic enrichment analysis to identify metabolic pathways that may be related to AIT. This study used two libraries: the Kyoto Encyclopedia of Genes and Genomes (KEGG) database, and the Small Molecule Pathway Database (SMPDB).

## 3 Results

### 3.1 Exploration of the causal effect of immune cells and AIT

**Figure 2:**
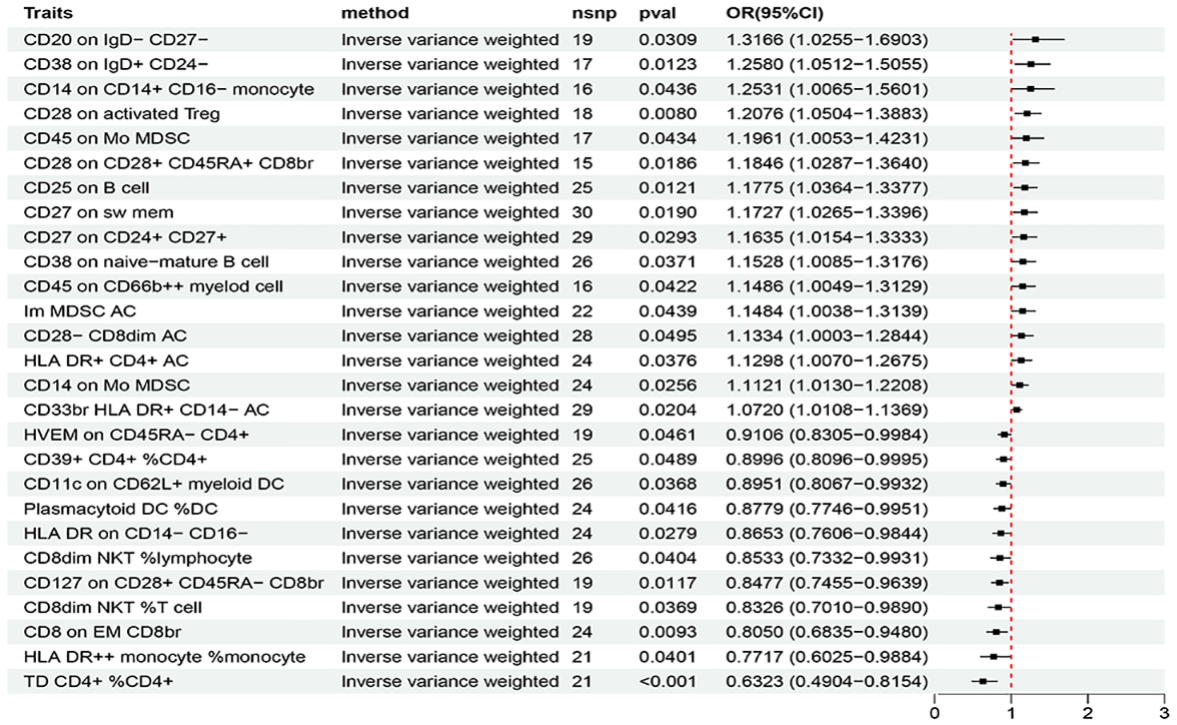
The forest plot to visualize effects of immune cell on the risk of AIT (IVW method).

**Figure 3:**
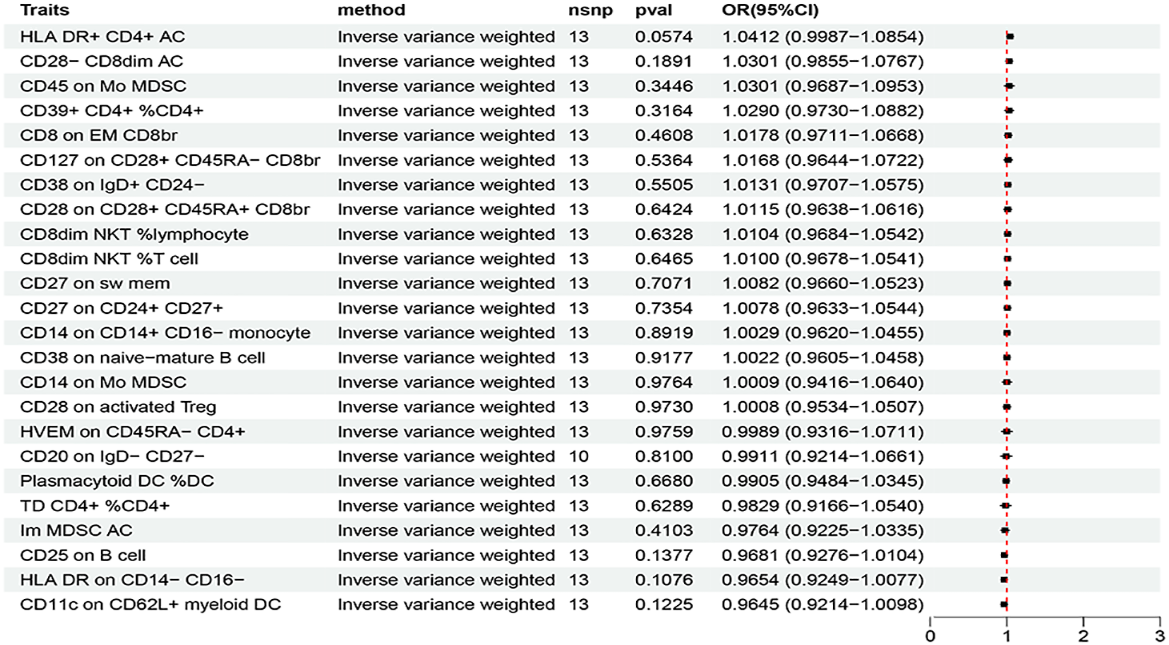
The forest plot to visualize effects of AIT on immune cells (IVW method).

Based on the criteria for selecting IVs, a total of 3 to 753 independent IVs were determined to investigate 731 immune cell traits. To explore the causal effects of immune cells on AIT, two-sample MR analysis was performed, and the IVW method was used as the main analysis. At the significance of 0.05, 27 suggestive immune cells were identified to be significantly associated with AIT at least in the IVM method, including 16 with risk effects (OR > 1) and 11 with protective effects (OR < 1) (**Figure 2**). Seventeen immune cells with risk effects on AIT onset fell into the B cell panel (including CD20 on IgD- CD27-, CD38 on IgD+ CD24-, CD25 on B cell, CD27 on sw mem, CD27 on CD24+ CD27+, CD38 on naive-mature B cell), the Myeloid cell panel (including CD45 on Mo MDSC, CD45 on CD66b++ myelod cell, Im MDSC AC, CD14 on Mo MDSC, CD33br HLA DR+ CD14- AC), the Treg panel (including CD28 on activated Treg, CD28 on CD28+ CD45RA+ CD8br, CD28- CD8dim AC), the Monocyte panel (CD14 on CD14+ CD16- monocyte), and the TBNK panel (HLA DR+ CD4+ AC) (**Figure 2**). Similar results were observed by using four more methods (**Supplementary Figure S1**; **Supplementary Table S1**). Eleven suggestive immune cells with protective effects on AIT onset fell into the Maturation stages of T cell panel (including TD CD4+ %CD4+, CD8 on EM CD8br, HVEM on CD45RA- CD4+), the TBNK panel (including HLA DR++ monocyte %monocyte, CD8dim NKT %T cell, CD8dim NKT %lymphocyte), the Treg panel (including CD127 on CD28+ CD45RA- CD8br, CD39+ CD4+ %CD4+), the DC panel (including Plasmacytoid DC %DC, CD11c on CD62L+ myeloid DC), and the Monocyte panel (HLA DR on CD14- CD16-) (**Figure 2**). Similar results were observed by using four more methods (**Supplementary Figure S1**; **Supplementary Table S1**). Characteristics of significant SNPs with genome-wide associations for immune cells on AIT were summarized in **Supplementary Table S2**. Additionally, both the intercept of MR-Egger and the global test of MR-PRESSO were used to evaluate the possibility of horizontal pleiotropy, and Cochran’s Q test was used to evaluate the possibility of heterogeneity. Horizontal pleiotropy was evaluated by both MR-EGGER and MR-PRESSO methods (**Supplementary Table S3**). Heterogeneity was suggested in the causal associations of AIT with HLA DR++ monocyte %monocyte, HLA DR+ CD4+ AC, CD20 on IgD- CD27-, HLA DR on CD14- CD16-, and CD45 on Mo MDSC (**Supplementary Table S4**). Reverse causal associations of AIT with immune cells were assessed as well. Based on the criteria for selecting IVs, 14 IVs for AIT (as exposure) were preserved for further analysis. Out of the 27 above-identified immune cells, no reverse causality was found for 24 immune cells, as revealed by our MR analysis (**Figure 3**; **Supplementary Table S5**), which were subsequently subjected to the mediation analysis. AIT was identified as a protective factor for three immune cells including HLA DR++ monocyte %monocyte (OR = 0.9377, 95% CI = 0.8979-0.9793, P = 0.0037), CD33br HLA DR+ CD14- AC (OR = 0.9381, 95% CI = 0.8869-0.9924, P = 0.0261), and CD45 on CD66b++ myelod cell (OR = 0.9313, 95% CI = 0.8760-0.9902, P = 0.0228) (**Supplementary Table S6**). No horizontal pleiotropy or heterogeneity was indicated as revealed by sensitivity analyses (**Supplementary Table S7**).

### 3.2 MR validation

A GWAS dataset of HT (ebi-a-GCST90018855), which incorporated meta-analyses of the UK Biobank and FinnGen data to improve the resolution of the genomic map of human diseases (23), was also used for MR validations. At the significance of 0.05, 31 suggestive immune cells were identified to be significantly associated with HT at least in the IVM method, including 12 with risk effects (OR > 1) and 19 with protective effects (OR < 1) (**Supplementary Figure S2**). Immune cells with risk effects on HT onset fell into the Maturation stages of T cell panel (including CD45RA on naive CD8br, CM DN (CD4-CD8-) %DN, HVEM on CD45RA- CD4+), B cell panel (CD25 on transitional), the Treg panel (including Resting Treg %CD4, CD39+ CD8br AC, CD28+ CD45RA+ CD8br %T cell, CD28 on activated & secreting Treg, CD28 on resting Treg), the TBNK panel (HLA DR++ monocyte %monocyte), and the DC panel (CD62L on monocyte, CCR2 on CD62L+ myeloid DC) (**Supplementary Table S8**). Immune cells with protective effects on HT onset fell into the Maturation stages of T cell panel (including Naive CD8br %T cell, EM CD8br %CD8br, CD45RA+ CD8br %CD8br, CD3 on CM CD8br), B cell panel (including IgD+ CD38dim %B cell, Sw mem %lymphocyte, CD24 on IgD- CD38dim, CD25 on memory B cell, CD25 on unsw mem), the Treg panel (including CD3 on CD45RA+ CD4+, CD3 on CD39+ CD8br, CD3 on CD4 Treg, CD3 on resting Treg), the TBNK panel (including CD8dim NKT AC, CD3 on HLA DR+ T cell, CD16-CD56 on NKT), and the DC panel (including CD62L- plasmacytoid DC %DC, CD86 on CD62L+ myeloid DC, CD127 on CD45RA- CD4 not Treg) (**Supplementary Table S8**). Similar results were observed by using four more methods (**Supplementary Figure S2**, **Supplementary Table S8**). Characteristics of significant SNPs with genome-wide associations for immune cells on HT were summarized in **Supplementary Table S9**. Possibilities of horizontal pleiotropy and heterogeneity were examined accordingly. Horizontal pleiotropy was suggested in the causal associations of HT with HLA DR++ monocyte %monocyte (**Supplementary Table S10**), and heterogeneity was suggested in HLA DR++ monocyte %monocyte and Resting Treg %CD4 (**Supplementary Table S11**). Reverse causal associations of HT with immune cells were assessed as well (**Supplementary Tables S12, S13**). Based on the criteria for selecting IVs, 11 IVs for HT (as exposure) were preserved for further analysis. As a result, HT was identified as a protective factor on HVEM on CD45RA- CD4+ (OR = 0.7067, 95% CI = 0.5626-0.8877, P = 0.0028) (**Supplementary Table S13**). No horizontal pleiotropy or heterogeneity was indicated as revealed by sensitivity analyses (**Supplementary Table S14**).

In a comparison of results of the MR analyses using AIT (FinnGen) and HT (ebi-a-GCST90018855) datasets, two identical immune cells: HLA DR++ monocyte %monocyte and HVEM on CD45RA- CD4+ were suggested to be involved in the onset of AIT/HT. However, as the horizontal pleiotropy, heterogeneity, or reverse causality was likely involved, neither was subjected to further mediation analysis. Though not identical, closely related immune cell traits were independently identified by the two outcome datasets (**Figure 2**; **Supplementary Figure S2**). Specifically by using the IVW method, in the Treg panel CD28 on CD28+ CD45RA+ CD8br (OR = 1.1846, 95% CI = 1.0287-1.3640, P = 0.0186), and CD28 on activated Treg (OR = 1.2076, 95% CI = 1.0504-1.3883, P = 0.0080) were risk factors for AIT, while CD28+ CD45RA+ CD8br %T cell (OR = 1.0049, CI = 1.0002-1.0095, P = 0.0410), CD28 on activated & secreting Treg (OR = 1.0320, 95% CI = 1.0035-1.0613, P = 0.0273), and CD28 on resting Treg (OR = 1.0808, 95% CI = 1.0053-1.1621, P =0.0355) were also identified as risk factors for HT; in the Maturation stages of T cell panel, CD8 on EM CD8br (OR = 0.8050, 95% CI = 0.6835-0.9480, P = 0.0093) was a protective factor for AIT, while EM CD8br %CD8br (OR = 0.9710, 95% CI = 0.7010-0.9890, P = 0.0042) was also identified as a protective factor for HT; in the TBNK panel CD8dim NKT %T cell (OR = 0.8326, 95% CI = 0.6835-0.9480, P = 0.0369), and CD8dim NKT %lymphocyte (OR = 0.8533, 95% CI = 0.7332-0. 0.9931, P = 0.0404) were protective factors for AIT, while CD8dim NKT AC (OR = 0.9314, 95% CI = 0.8858-0.9794, P = 0.0056) was also identified as a protective factor for HT; in the DC panel CD11c on CD62L+ myeloid DC (OR = 0.8951, 95% CI = 0.8067-0.9932, P = 0.0368) was a protective factor for AIT, while CD86 on CD62L+ myeloid DC (OR = 0.9605, 95% CI = 0.9247-0.9979, P = 0.0386) and was identified as a protective factor for HT; in the B cell panel CD25 on B cell (OR = 1.1775, 95% CI = 1.0364-1.3377, P = 0.0121) was a risk factor for AIT, while CD25 on transitional B cell (OR = 1.0694, 95% CI = 1.0192-1.1221, P = 0.0062) and was identified as a risk factor for HT (**Figure 2**; **Supplementary Figure S2**).

### 3.3 Association of metabolites with AIT

**Figure 4:**
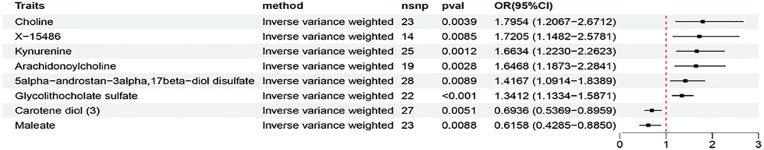
The forest plot to visualize effects of metabolites on AIT (IVW method).

Based on the criteria for selecting IVs, a total of 12 to 93 independent IVs were determined to investigate 1400 metabolite traits. At the significance of 0.01, 10 suggestive metabolites, including 8 metabolites (choline, X-15486, kynurenine, arachidonoylcholine, vanillic alcohol sulfate, sphingomyelin, 5alpha-androstan-3alpha,17beta-diol disulfate, glycolithocholate sulfate) with risk effects and 2 metabolites (carotene diol (3), maleate) with protective effects, were identified to be significantly associated with AIT at least in the IVM method (**Figure 4**; **Supplementary Figure S3**). In MR validation analysis using the HT dataset, 9 suggestive metabolites were identified to be significantly associated with HT at least in the IVM method (**Supplementary Figure S4**). The estimation directions of all five methods, IVW, MR-Egger, weighted median, simple mode, and weighted mode were consistent (**Supplementary Figures S3, S4**; **Supplementary Tables S15**, **S19**). Characteristics of significant SNPs with genome-wide associations for metabolites on AIT or HT were summarized in **Supplementary Tables S16**, **S20**. No heterogeneity (**Supplementary Tables S17**, **S21**) or horizontal pleiotropy (**Supplementary Tables S18**, **S22**) was indicated as revealed by sensitivity analyses.

At the significance of 0.05, an identical metabolite was identified independently with both AIT and HT datasets: 5alpha-androstan-3alpha,17beta-diol disulfate was identified as suggestive risk factor for AIT (OR=1.4167,95% CI = 1.0914-1.8389, P = 0.0089) and HT (OR=1.0781,95% CI = 1.0065-1.1548, P = 0.0319) (**Supplementary Tables S15**, **S19**). No heterogeneity (**Supplementary Tables S17**, **S21**) or horizontal pleiotropy (**Supplementary Tables S18**, **S22**) was indicated. Besides, a total of 58 suggestive metabolic traits (including 47 metabolites and 11 metabolic ratios) were identified to be significantly associated with AIT, at least in the IVM method (P < 0.05). Enrichment analysis using the identified AIT-associated metabolites revealed ‘Tryptophan Metabolism’ as the top 1 enriched metabolic set possibly related to AIT (**Supplementary Figure S5**).

### 3.4 Association of immune cells with metabolites

**Figure 5:**
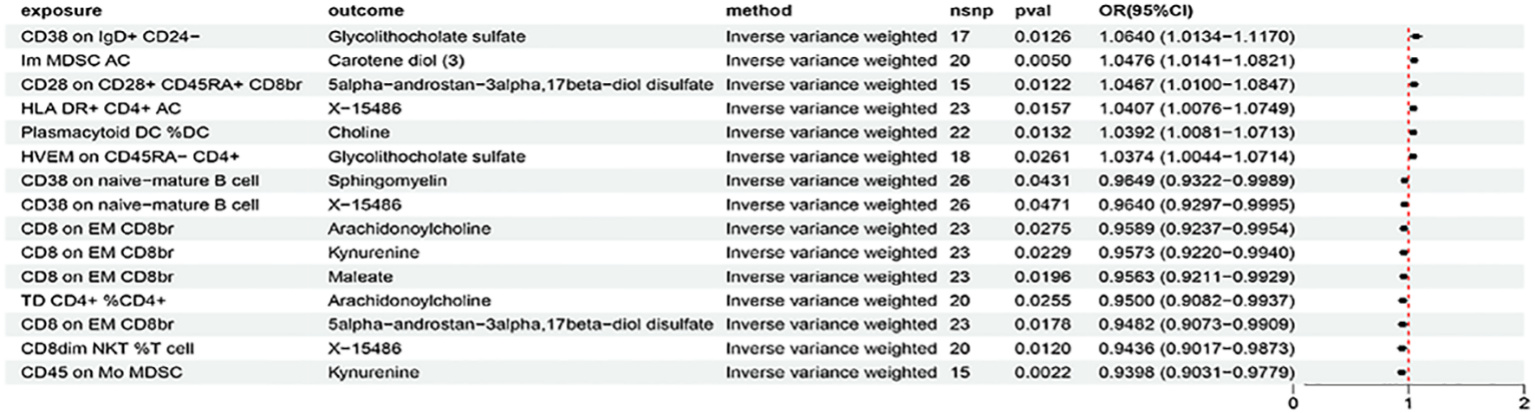
The forest plot to visualize effects of immune cells on metabolites (IVW method).

The above identified AIT-associated immune cells were further evaluated for their causal effects on the levels of metabolites that were causally associated with AIT. At the significance of 0.05, 15 suggestive causal associations involving 11 AIT-associated immune cells and 9 AIT-associated metabolites were identified at least in the IVM method (**Figure 5**). Specifically by using the IVW method, CD38 on IgD+ CD24- was associated with increased glycolithocholate sulfate levels (OR = 1.0640, 95% CI = 1.0134-1.1170, P = 0.0126), CD28 on CD28+ CD45RA+ CD8br was associated with increased 5alpha-androstan-3alpha,17beta-diol disulfate levels (OR = 1.0467, 95% CI = 1.0100-1.0847, P = 0.0122), HLA DR+ CD4+ AC was associated with increased X-15486 levels (OR = 1.0407, 95% CI = 1.0076-1.0749, P = 0.0157), TD CD4+ %CD4+ was associated with decreased arachidonoylcholine levels (OR = 0.9500, 95% CI = 0.9082-0.9937, P = 0.0255), CD8dim NKT %T cell was associated with decreased X-15486 levels (OR = 0.9435, 95% CI = 0.9017-0.9873, P = 0.0120), and CD8 on EM CD8br was associated with decreased levels of kynurenine (OR = 0.9573, 95% CI = 0.9220-0.9940, P = 0.0229), 5alpha-androstan-3alpha,17beta-diol disulfate (OR = 0.9482, 95% CI = 0.9073-0.9909, P = 0.0178), and arachidonoylcholine (OR = 0.9589, 95% CI = 0.9237-0.9954, P = 0.0275). Similar results were observed by using four more methods (**Supplementary Figure S6**; **Supplementary Table S23**). Characteristics of significant SNPs with genome-wide associations for immune cells on metabolites were summarized in **Supplementary Table S24**. No heterogeneity (**Supplementary Table S25**) or horizontal pleiotropy (**Supplementary Table S26**) was indicated as revealed by sensitivity analyses.

### 3.5 Proportion of the association between immune cells and AIT mediated by metabolites

**Figure 6:**
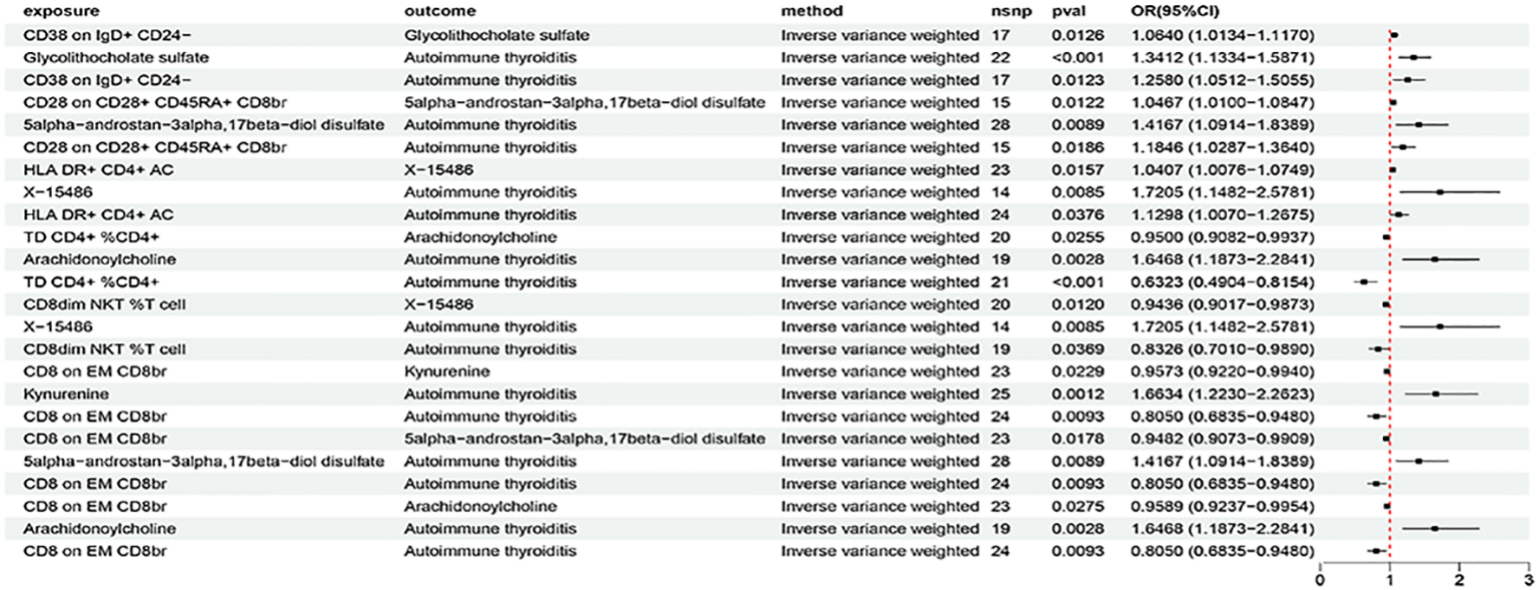
The forest plot to visualize effects of immune cells on AIT through the mediation of metabolites (IVW method).

In summary, we analyzed metabolites as a mediator of the pathway from immune cells to AIT using the IVW method as the main analysis (**Figure 6**). We found that CD38 on IgD+ CD24- was associated with increased glycolithocholate sulfate levels, which in turn was associated with an increased risk of AIT. Glycolithocholate sulfate accounted for 7.93% of the increased risk of AIT associated with CD38 on IgD+ CD24- (proportion mediated: 7.93%; 95% CI = -13.6%−29.5%). CD28 on CD28+ CD45RA+ CD8br was associated with increased 5alpha-androstan-3alpha,17beta-diol disulfate levels, which in turn was associated with an increased risk of AIT. 5alpha-androstan-3alpha,17beta-diol disulfate accounted for 9.39% of the increased risk of AIT associated with CD28 on CD28+ CD45RA+ CD8br (proportion mediated: 9.39%; 95% CI = -44.3%−63%). HLA DR+ CD4+ AC was associated with increased X-15486 levels, which in turn was associated with an increased risk of AIT. X-15486 accounted for 17.7% of the increased risk of AIT associated with HLA DR+ CD4+ AC (proportion mediated: 17.7%; 95% CI = -162%−198%). TD CD4+ %CD4+ was associated with decreased arachidonoylcholine levels, while arachidonoylcholine was associated with an increased risk of AIT. Arachidonoylcholinethus accounted for 5.58% of the decreased risk of AIT associated with TD CD4+ %CD4+ (proportion mediated: 5.58%; 95% CI = 41.2%−-30%). CD8dim NKT %T cell was associated with decreased X-15486 levels, while X-15486 was associated with an increased risk of AIT. X-15486 thus accounted for 17.2% of the decreased risk of AIT associated with CD8dim NKT %T cell (proportion mediated: 17.2%; 95% CI = 137%−-103%). CD8 on EM CD8brwas associated with decreased levels of arachidonoylcholine, kynurenine, and 5alpha-androstan-3alpha,17beta-diol disulfate levels; all three metabolites was associated with an increased risk of AIT. Kynurenine accounted for 10.2% of the decreased risk of AIT associated with CD8 on EM CD8br (proportion mediated: 10.2%; 95% CI = 82.4%−-61.9%); arachidonoylcholine accounted for 9.66% of the decreased risk of AIT associated with CD8 on EM CD8br (proportion mediated: 9.66%; 95% CI = 84.9%− -65.6%); and 5alpha-androstan-3alpha,17beta-diol disulfate accounted for8.54% of the decreased risk of AIT associated with CD8 on EM CD8br (proportion mediated: 8.54%; 95% CI = 50.4%−-33.4%). Similar results were observed by using four more methods (**Supplementary Figure S7**; **Supplementary Table S27**). Suggestive heterogeneity (by Cochran’s Q test) (**Supplementary Table S28**) and pleiotropy (by MR-PRESSO) (**Supplementary Table S29**) was indicated in the association of HLA DR+ CD4+ AC with AIT, otherwise, detailed information from the sensitivity analysis proved the robustness of the causal associations observed in the causal associations. Moreover, scatter plots (**Supplementary Figures S8**–**S10**), funnel plots (**Supplementary Figures S11**–**S13**), and forest plots for MR leave-one-out sensitivity analysis (**Supplementary Figures S14**–**S16**) were also generated to visualize the overall stability in the results.

## 4 Discussion

Based on large publicly available genetic data, we explore causal associations between 731 immune cell traits and AIT through potential mediation of 1400 metabolic traits. We identified 27 immune cells and 10 metabolites to be causally associated with AIT (at least in the IVM method), among which 5 immune cells (including CD38 on IgD+ CD24-, CD28 on CD28+ CD45RA+ CD8br, HLA DR+ CD4+ AC, TD CD4+ %CD4+, and CD8 on EM CD8br) were found to affect the onset of AIT partially through the mediation of metabolites (including glycolithocholate sulfate, 5alpha-androstan-3alpha,17beta-diol disulfate, arachidonoylcholine, X-15486, and kynurenine). The results suggest that metabolites were able to, at least partially, mediate the causal relationship between immune cells and AIT. To our knowledge, this is the first study that thoroughly explored potential causal associations among immune cells, metabolites, and AIT.

We identified that CD28 on CD28+ CD45RA+ CD8br was associated with increased 5alpha-androstan-3alpha,17beta-diol disulfate levels, which in turn was associated with an increased risk of AIT. In addition to CD28 on CD28+ CD45RA+ CD8br, CD28 on activating Treg was also a risk factor for AIT. Similarly, CD28+ CD45RA+ CD8br %T cell, CD28 on activated & secreting Treg, and CD28 on resting Treg were all identified as risk factors in MR validation using the HT dataset (ebi-a-GCST90018855). This is consistent with the previous finding that CD28+ CD45RA+ CD8br was a suggestive risk factor for AIT (24, 39). CD28 is a well-known key co-stimulatory molecule expressed on T cells, required for T cell activation. The expression of CD28 on T cells was coincidently affected by genetic variation in the *BACH2* gene region (40). A single fine-mapped variant (rs72928038[A]) of *BACH2* is associated with increased CD28 expression on CD45RA+ cells and overlapped with increased risk for AIT (40). Another variant in *BACH2* positively associated with the same immune cell traits also overlapped with increased risk for type 1 diabetes (T1D) and systemic lupus erythematosus (SLE) (41). These findings link genetic variations in *BACH2* to CD28 regulation, indicating their synergistic role in directing T cell fate toward inflammatory or autoimmune status. Furthermore, CD28 has been predicted as a primary drug target for AIT based on three considerations: its expression can be influenced by the *cis*-acting signal of *BACH2* underlying coincident associations; its pharmacological modulation might reproduce a therapeutic protective effect on AIT; and the anti-CD28 drug was already developed for SLE (24). On the other hand, 5alpha-androstan-3alpha,17beta-diol disulfate is a major androstane sulfate, which was also identified as a risk factor in the MR validation using the HT dataset. Through constitutive androstane receptor (CAR), metabolites such as androstane sulfates possibly participate in the regulation of intestinal mucosal homeostasis and inflammation (42). This metabolite has also been suggested to play an important role in the regulation of the hypothalamo-pituitary–adrenal (HPA) axis (43). The specific regulatory role of 5alpha-androstan-3alpha,17beta-diol disulfate in CD28+ Treg and consequentially in their associations with AIT needs to be further explored.

CD8 on EM CD8br was a protective factor of AIT, and is associated with decreased levels of kynurenine, arachidonoylcholine, and 5alpha-androstan-3alpha,17beta-diol disulfate levels; all three metabolites were associated with increased risk of AIT. Similarly, EM CD8br %CD8br was also identified as a protective factor in MR validation using the HT dataset. CD8 on EM CD8br or EM CD8br %CD8br is an effector memory T-cell (T_EM_) trait from the panel of maturation stages of T cell (24). CD8+ T cells of memory phenotype and function can arise in response to self-peptide and/or in a lymphopenic environment in response to cytokines that trigger homeostatic proliferation (“virtual” and “innate” memory) (44). It has been proposed that T_EM_ are specialized to handle infections arising within peripheral organs due to their cytotoxicity and ability to localize to tissues (44). T_EM_ cells likely drive the persistence of autoimmune diseases because of their ready effector functionality and relative longevity. As shown in studies of chronic infections, persistent antigen increases the pool of T_EM_ cells, which might be the case in autoimmune disease settings with persistence of self-antigens (45). A higher percentage of T_EM_ was reported in the cerebrospinal fluid (CSF) lymphocytes of patients with various inflammatory diseases compared with non-inflammatory controls (46). However, the role of T_EM_ in AIT was yet studied. Our enrichment analysis using AIT-associated metabolites also revealed ‘Tryptophan Metabolism’ as a major enriched metabolic set. Tryptophan (Trp) is an essential amino acid, which is utilized via the dominant kynurenine pathway (up to 95% of Trp metabolism). Upon immune stimulation, the so called ‘kynurenine switch’ snaps into action, and most Trp molecules enter immunocompetent cells and are metabolized therein via the kynurenine pathway (47). Kynurenine binds to the aryl hydrocarbon receptor (AHR) in multiple immune cell types, leading to immune regulation (19, 48). It has been shown that after immune stimulation, immune cells generate high levels of various intracellular Trp metabolites (49), which in turn is involved in the induction of the synthesis of cytoskeletal proteins and regulation of immune cell motility (50). Indoleamine 2,3-dioxygenase 1 (IDO1), the key enzyme of kynurenine pathway, is one of the major regulators of the function of immune cells, controlling the balance between stimulation and suppression of the immune system at sites of local inflammation and thus is relevant to a wide range of autoimmune and inflammatory diseases (19). Significant attention has been paid to the immunoregulatory role of IDO1 in the most prevalent, organ-specific autoimmune endocrinopathies such as T1D and AIT (19, 48). It was reported that the serum kynurenine metabolite profile was dysregulated in young women with AIT and could serve as a new predictor of AIT risk (19). Numerous studies have been devoted to the role of metabolites of the kynurenine pathway of Trp metabolism in immune regulation, as well as to the analysis of the effects of kynurenine on the activity of immunocompetent cells (48). These works showed that the disruption of kynurenine metabolism is one of the key mechanisms of autoimmune provocations (51). Modulation of the kynurenine pathway reversed the progression of experimental autoimmune encephalomyelitis (52), indicating a potential prophylactic and therapeutic effect of the kynurenine pathway on autoimmune diseases. How kynurenine links the immune cells, particularly T_EM_, and AIT merits further investigation.

Choline (a component of the majority of phospholipids in membranes of mammalian cells), arachidonoylcholine (acylated derivatives of choline), and sphingomyelin (a dominant sphingolipid in membranes of mammalian cells) were all risk factors for AIT revealed by our analysis. Cholines acylated with unsaturated fatty acids are a recently discovered family of endogenous lipids (53, 54). These are acylated derivatives of choline. Arachidonoylcholine, also known as choline arachidonate, belongs to the class of acylcholine (53, 54). Choline has been found to affect dry eye syndrome and autoimmune diseases (55). Choline modulates the function of inflammatory response of immune cells (56). Impaired choline uptake and metabolism modulate macrophage IL- 1β and IL-18 production, and its kinase inhibitors ameliorated acute and chronic models of IL-1β-dependent inflammation (57). Sphingomyelin is a prospective metabolic immune checkpoint for natural killer cells and is important for directing immune responses (58, 59). It is also known that lipid metabolism has a complex relationship with AIT: patients with AIT are prone to lipid metabolism disorder, and the serum thyroid hormone level has a close correlation with blood lipid metabolism and inflammatory factors (60). Phospholipids influence the pathogenesis of HT, and fatty acid degradation and lysine degradation pathways have an impact on different clinical stages of HT (18). TD CD4+ %CD4+ that features the percentage of terminally differentiated CD4+ T cells (24) was also found to affect the onset of AIT partially through the mediation of arachidonoylcholine in this study. The precise mechanisms by which arachidonoylcholine and/or choline link the pathway from immune cells to AIT need more in-depth studies in the future.

In the B cell panel, CD38 on IgD+ CD24- was found to be associated with increased glycolithocholate sulfate levels, which in turn was associated with an increased risk of AIT. CD38 on IgD+ CD24- represents plasmablasts that rapidly produced and short-lived effector cells of the early antibody response, to the contrast of plasma cells that are long-lived mediators of the lasting humoral immunity (61). Short-lived plasmablasts can be associated with disease flares and increased autoantibody levels (62). Plasmablasts that produce autoantibodies can be pathogenic or regulatory. Comparing these two types of plasmablasts may help researchers understand the mechanisms of autoantibody-related autoimmunological diseases (62). Plasmablasts might be as promising a therapeutic target in autoimmune diseases as the plasma cells (63). Glycolithocholic acid 3-sulfate is a cholic acid derivative and a metabolite of glycolithocholic acid, which belongs to secondary bile acids (BAs). Cholic acid and chenodeoxycholic acid are the primary bile acids formed in humans. The action of intestinal bacterial flora on primary bile acids results in the formation of secondary BAs (64). Secondary BAs contribute to the differentiation of Tregs, thus participating in the regulation of host immune responses (65). Via the secondary BAs, a pan-genomic biliary network interaction between hosts and their bacterial symbionts can control host immunological homeostasis (65). Evidence also shows that BAs could regulate the differentiation of Th17 through its surface receptor RORγt (66). There is a growing consensus that BAs have both pro- and anti-inflammatory actions through different nuclear and cell surface BA receptors in the intestine, as well as the liver (67). Whether this biliary network interaction could participate in the pathogenesis of AIT through its resulting metabolites raises an interesting question. In addition to plasmablasts, CD27 on sw mem, representing the immune trait of switched memory B cells, was also identified as a risk factor of AIT. It was previously reported that a signal led by rs1883832[T] in the 5’ untranslated region of *CD40* increases the expression of CD27 on memory B cell subsets, which overlaps with increased risk of various autoimmune diseases such as multiple sclerosis (MS), inflammatory bowel disease (IBD), Crohn’s disease, and SLE (24).

HLA DR+ CD4+ AC and CD8dim NKT %T cell were both identified to affect AIT through the same unknown metabolite X-15486. HLA DR+ CD4+ AC was identified to be a risk factor for AIT. HLA-DR+ CD4 T cells could be a useful marker for identifying effector T cells and monitoring immune responses in many infection and vaccination models (68). *HLA-DR* is a well-recognized susceptibility gene associated with AIT (69). Mechanistically, the presence of an arginine at position 74 elicits a significant structural change in the peptide binding pocket of *HLA-DR*, potentially affecting the binding of pathogenic thyroidal peptides (69). Future therapeutic interventions may attempt to block or modulate pathogenic peptide presentation by *HLA-DR*. CD8dim NKT %T cell and CD8dim NKT %lymphocyte were both identified to be associated with decreased risk of AIT; similarly, CD8dim NKT AC was also a protective factor in MR validation using the HT dataset. It has been reported that CD8^+^NKT-like cells suppressed T-cell responses through elimination of dendritic cells in an antigen-specific manner (70). Such antigen-specific downregulation may provide an active and precise method for constraining an excessive immune response and avoiding bypass suppression of necessary immune responses to other antigens (70). Whether CD8dim NKT or CD8^+^NKT-like cells act in a similar manner in AIT requires further investigation.

This study conducted two-sample and mediation MR analyses based on the published results of large GWAS cohorts, so it has relatively high statistical efficiency. The conclusions of this study are based on genetic instrumental variables, and causal inference is made using a variety of MR analysis methods. Our results are robust and were not confounded by horizontal pleiotropy and other factors. Hopefully, our study provides new insights into the integration of immune cells and metabolites for further exploration of the biological mechanisms of AIT, and some guidance for potential therapeutic strategies for AIT. Nevertheless, there were several limitations in our study. First, our analysis was performed using the European population, which limits its prevalence. Second, the numbers of individuals in the current GAWS datasets of immune cell traits and metabolites were relatively small and it is hoped that larger GWAS data will be available for validation in the future. Third, even when multiple sensitivity analyses are performed, horizontal pleiotropy cannot be fully assessed. Fourth, due to the lack of individual information, we cannot conduct further stratified analysis of the population. Fifth, we used a relatively looser threshold to evaluate the results, which may increase some false positives while simultaneously enabling a more comprehensive assessment of the strong association between the immune or metabolic profiles with AIT. Sixth, some immune cell traits or metabolites identified in this study have not been fully elucidated concerning their functions and mechanisms in AIT, which limits our interpretation of the MR analysis results. Lastly, although the MR method is effective in evaluating the causal relationship between exposure factors and outcomes, the results needs to be further validated based on more experimental and clinical studies.

## Data availability statement

The original contributions presented in the study are included in the article/**Supplementary Material**. Further inquiries can be directed to the corresponding authors.

## Author contributions

YC: Investigation, Methodology, Software, Visualization, Writing – original draft. BJ: Data curation, Investigation, Methodology, Writing – review & editing. CQ: Data curation, Formal analysis, Methodology, Writing – review & editing. CJ: Formal analysis, Software, Validation, Writing – review & editing. CZ: Data curation, Formal analysis, Methodology, Writing – review & editing. YW: Investigation, Validation, Visualization, Writing – review & editing. FC: Resources, Software, Writing – review & editing. XS: Conceptualization, Resources, Writing – review & editing. LS: Conceptualization, Supervision, Writing – review & editing. YL: Conceptualization, Funding acquisition, Supervision, Validation, Writing – original draft, Writing – review & editing.

## References

[1] Ragusa F Fallahi P Elia G Gonnella D Paparo SR Giusti C. Hashimotos' thyroiditis: Epidemiology, pathogenesis, clinic and therapy. Best Pract Res Clin Endocrinol Metab. (2019) 33:101367. doi: 10.1016/j.beem.2019.101367 31812326

[2] Weetman AP. An update on the pathogenesis of Hashimoto's thyroiditis. J Endocrinol Invest. (2021) 44:883–90. doi: 10.1007/s40618-020-01477-1 PMC8049926 33332019

[3] Janyga S Marek B Kajdaniuk D Ogrodowczyk-Bobik M Urbanek A Buldak L. CD4+ cells in autoimmune thyroid disease. Endokrynol Pol. (2021) 72:572–83. doi: 10.5603/EP.a2021.0076 34647609

[4] Lechner MG Zhou Z Hoang AT Huang N Ortega J Scott LN. Clonally expanded, thyrotoxic effector CD8(+) T cells driven by IL-21 contribute to checkpoint inhibitor thyroiditis. Sci Transl Med. (2023) 15:eadg0675. doi: 10.1126/scitranslmed.adg0675 37196065 PMC10227862

[5] Shao S Yu X Shen L. Autoimmune thyroid diseases and Th17/Treg lymphocytes. Life Sci. (2018) 192:160–65. doi: 10.1016/j.lfs.2017.11.026 29158050

[6] Luty J Ruckemann-Dziurdzinska K Witkowski JM Bryl E. Immunological aspects of autoimmune thyroid disease - Complex interplay between cells and cytokines. Cytokine. (2019) 116:128–33. doi: 10.1016/j.cyto.2019.01.003 30711852

[7] Zhang J Zhao L Gao Y Liu M Li T Huang Y. A classification of Hashimoto's thyroiditis based on immunohistochemistry for IgG4 and IgG. Thyroid. (2014) 24:364–70. doi: 10.1089/thy.2013.0211 23992023

[8] Liu L Yu Y Chen L Zhang Y Lu G Gao Y. Clinical differences between IgG4 Hashimoto's thyroiditis and primary thyroid lymphoma. Eur Thyroid J. (2022) 11:e210144. doi: 10.1530/ETJ-21-0144 35521776 PMC9175605

[9] D'Ippolito S Ticconi C Tersigni C Garofalo S Martino C Lanzone A. The pathogenic role of autoantibodies in recurrent pregnancy loss. Am J Reprod Immunol. (2020) 83:e13200. doi: 10.1111/aji.13200 31633847

[10] Ticconi C Giuliani E Veglia M Pietropolli A Piccione E Di Simone N. Thyroid autoimmunity and recurrent miscarriage. Am J Reprod Immunol. (2011) 66:452–9. doi: 10.1111/aji.2011.66.issue-6 21623997

[11] Dai Z Ramesh V Locasale JW. The evolving metabolic landscape of chromatin biology and epigenetics. Nat Rev Genet. (2020) 21:737–53. doi: 10.1038/s41576-020-0270-8 PMC8059378 32908249

[12] Michalek RD Gerriets VA Jacobs SR Macintyre AN MacIver NJ Mason EF. Cutting edge: distinct glycolytic and lipid oxidative metabolic programs are essential for effector and regulatory CD4+ T cell subsets. J Immunol. (2011) 186:3299–303. doi: 10.4049/jimmunol.1003613 PMC3198034 21317389

[13] Saucillo DC Gerriets VA Sheng J Rathmell JC Maciver NJ. Leptin metabolically licenses T cells for activation to link nutrition and immunity. J Immunol. (2014) 192:136–44. doi: 10.4049/jimmunol.1301158 PMC3872216 24273001

[14] De Rosa V La Cava A Matarese G. Metabolic pressure and the breach of immunological self-tolerance. Nat Immunol. (2017) 18:1190–96. doi: 10.1038/ni.3851 29044230

[15] Otero M Lago R Gomez R Lago F Dieguez C Gomez-Reino JJ. Changes in plasma levels of fat-derived hormones adiponectin, leptin, resistin and visfatin in patients with rheumatoid arthritis. Ann Rheum Dis. (2006) 65:1198–201. doi: 10.1136/ard.2005.046540 PMC1798289 16414972

[16] Ziegler JF Bottcher C Letizia M Yerinde C Wu H Freise I. Leptin induces TNFalpha-dependent inflammation in acquired generalized lipodystrophy and combined Crohn's disease. Nat Commun. (2019) 10:5629. doi: 10.1038/s41467-019-13559-7 31822667 PMC6904732

[17] Tsoukalas D Fragoulakis V Sarandi E Docea AO Papakonstaninou E Tsilimidos G. Targeted metabolomic analysis of serum fatty acids for the prediction of autoimmune diseases. Front Mol Biosci. (2019) 6:120. doi: 10.3389/fmolb.2019.00120 31737644 PMC6839420

[18] Jiang X Zhao X Gu X Luo T Li P Wan C. Serum metabolomic analysis in patients with Hashimoto's thyroiditis. Front Endocrinol (Lausanne). (2022) 13:1046159. doi: 10.3389/fendo.2022.1046159 36619550 PMC9814722

[19] Krupa A Lebkowska A Kondraciuk M Kaminski KA Kowalska I. Alteration in kynurenine pathway metabolites in young women with autoimmune thyroiditis. Sci Rep. (2024) 14:6851. doi: 10.1038/s41598-024-57154-3 38514790 PMC10957988

[20] Song J Shan Z Mao J Teng W. Serum polyamine metabolic profile in autoimmune thyroid disease patients. Clin Endocrinol (Oxf). (2019) 90:727–36. doi: 10.1111/cen.13946 30725486

[21] Emdin CA Khera AV Kathiresan S. Mendelian randomization. JAMA. (2017) 318:1925–26. doi: 10.1001/jama.2017.17219 29164242

[22] Skrivankova VW Richmond RC Woolf BAR Yarmolinsky J Davies NM Swanson SA. Strengthening the reporting of observational studies in epidemiology using Mendelian randomization: the STROBE-MR statement. JAMA. (2021) 326:1614–21. doi: 10.1001/jama.2021.18236 34698778

[23] Sakaue S Kanai M Tanigawa Y Karjalainen J Kurki M Koshiba S. A cross-population atlas of genetic associations for 220 human phenotypes. Nat Genet. (2021) 53:1415–24. doi: 10.1038/s41588-021-00931-x PMC12208603 34594039

[24] Orru V Steri M Sidore C Marongiu M Serra V Olla S. Complex genetic signatures in immune cells underlie autoimmunity and inform therapy. Nat Genet. (2020) 52:1036–45. doi: 10.1038/s41588-020-0684-4 PMC8517961 32929287

[25] Sidore C Busonero F Maschio A Porcu E Naitza S Zoledziewska M. Genome sequencing elucidates Sardinian genetic architecture and augments association analyses for lipid and blood inflammatory markers. Nat Genet. (2015) 47:1272–81. doi: 10.1038/ng.3368 PMC4627508 26366554

[26] Chen Y Lu T Pettersson-Kymmer U Stewart ID Butler-Laporte G Nakanishi T. Genomic atlas of the plasma metabolome prioritizes metabolites implicated in human diseases. Nat Genet. (2023) 55:44–53. doi: 10.1038/s41588-022-01270-1 36635386 PMC7614162

[27] Genomes Project C Auton A Brooks LD Durbin RM Garrison EP Kang HM. A global reference for human genetic variation. Nature. (2015) 526:68–74. doi: 10.1038/nature15393 26432245 PMC4750478

[28] Burgess S Thompson SG Collaboration CCG. Avoiding bias from weak instruments in Mendelian randomization studies. Int J Epidemiol. (2011) 40:755–64. doi: 10.1093/ije/dyr036 21414999

[29] Burgess S Small DS Thompson SG. A review of instrumental variable estimators for Mendelian randomization. Stat Methods Med Res. (2017) 26:2333–55. doi: 10.1177/0962280215597579 PMC5642006 26282889

[30] Burgess S Thompson SG. Interpreting findings from Mendelian randomization using the MR-Egger method. Eur J Epidemiol. (2017) 32:377–89. doi: 10.1007/s10654-017-0255-x PMC5506233 28527048

[31] Bowden J Davey Smith G Haycock PC Burgess S. Consistent estimation in Mendelian randomization with some invalid instruments using a weighted median estimator. Genet Epidemiol. (2016) 40:304–14. doi: 10.1002/gepi.21965 PMC4849733 27061298

[32] Hartwig FP Davey Smith G Bowden J. Robust inference in summary data Mendelian randomization via the zero modal pleiotropy assumption. Int J Epidemiol. (2017) 46:1985–98. doi: 10.1093/ije/dyx102 PMC5837715 29040600

[33] Burgess S Dudbridge F Thompson SG. Combining information on multiple instrumental variables in Mendelian randomization: comparison of allele score and summarized data methods. Stat Med. (2016) 35:1880–906. doi: 10.1002/sim.6835 PMC4832315 26661904

[34] Bowden J Davey Smith G Burgess S. Mendelian randomization with invalid instruments: effect estimation and bias detection through Egger regression. Int J Epidemiol. (2015) 44:512–25. doi: 10.1093/ije/dyv080 PMC4469799 26050253

[35] Boehm FJ Zhou X. Statistical methods for Mendelian randomization in genome-wide association studies: A review. Comput Struct Biotechnol J. (2022) 20:2338–51. doi: 10.1016/j.csbj.2022.05.015 PMC9123217 35615025

[36] Carter AR Sanderson E Hammerton G Richmond RC Davey Smith G Heron J. Mendelian randomisation for mediation analysis: current methods and challenges for implementation. Eur J Epidemiol. (2021) 36:465–78. doi: 10.1007/s10654-021-00757-1 PMC8159796 33961203

[37] Cohen JF Chalumeau M Cohen R Korevaar DA Khoshnood B Bossuyt PM. Cochran's Q test was useful to assess heterogeneity in likelihood ratios in studies of diagnostic accuracy. J Clin Epidemiol. (2015) 68:299–306. doi: 10.1016/j.jclinepi.2014.09.005 25441698

[38] Verbanck M Chen CY Neale B Do R. Detection of widespread horizontal pleiotropy in causal relationships inferred from Mendelian randomization between complex traits and diseases. Nat Genet. (2018) 50:693–98. doi: 10.1038/s41588-018-0099-7 PMC6083837 29686387

[39] Jiang YJ Xiong YQ Huang T Xiao YX. Toenail and blood selenium mediated regulation of thyroid dysfunction through immune cells: a mediation Mendelian randomization analysis. Front Nutr. (2024) 11:1378969. doi: 10.3389/fnut.2024.1378969 38840695 PMC11150534

[40] Cooper JD Simmonds MJ Walker NM Burren O Brand OJ Guo H. Seven newly identified loci for autoimmune thyroid disease. Hum Mol Genet. (2012) 21:5202–8. doi: 10.1093/hmg/dds357 PMC3490518 22922229

[41] International Multiple Sclerosis Genetics C Beecham AH Patsopoulos NA Xifara DK Davis MF Kemppinen A. Analysis of immune-related loci identifies 48 new susceptibility variants for multiple sclerosis. Nat Genet. (2013) 45:1353–60. doi: 10.1038/ng.2770 PMC3832895 24076602

[42] Hudson GM Flannigan KL Erickson SL Vicentini FA Zamponi A Hirota CL. Constitutive androstane receptor regulates the intestinal mucosal response to injury. Br J Pharmacol. (2017) 174:1857–71. doi: 10.1111/bph.13787 PMC5446585 28320072

[43] Handa RJ Sharma D Uht R. A role for the androgen metabolite, 5alpha androstane 3beta, 17beta diol (3beta-diol) in the regulation of the hypothalamo-pituitary-adrenal axis. Front Endocrinol (Lausanne). (2011) 2:65. doi: 10.3389/fendo.2011.00065 22649380 PMC3355903

[44] Martin MD Badovinac VP. Defining memory CD8 T cell. Front Immunol. (2018) 9:2692. doi: 10.3389/fimmu.2018.02692 30515169 PMC6255921

[45] Devarajan P Chen Z. Autoimmune effector memory T cells: the bad and the good. Immunol Res. (2013) 57:12–22. doi: 10.1007/s12026-013-8448-1 24203440 PMC4067599

[46] Mullen KM Gocke AR Allie R Ntranos A Grishkan IV Pardo C. Expression of CCR7 and CD45RA in CD4+ and CD8+ subsets in cerebrospinal fluid of 134 patients with inflammatory and non-inflammatory neurological diseases. J Neuroimmunol. (2012) 249:86–92. doi: 10.1016/j.jneuroim.2012.04.017 22633193 PMC3391349

[47] Haq S Grondin JA Khan WI. Tryptophan-derived serotonin-kynurenine balance in immune activation and intestinal inflammation. FASEB J. (2021) 35:e21888. doi: 10.1096/fj.202100702R 34473368 PMC9292703

[48] Krupa A Kowalska I. The Kynurenine pathway-new linkage between innate and adaptive immunity in autoimmune endocrinopathies. Int J Mol Sci. (2021) 22:9879–916. doi: 10.3390/ijms22189879 34576041 PMC8469440

[49] Moffett JR Arun P Puthillathu N Vengilote R Ives JA Badawy AA. Quinolinate as a marker for kynurenine metabolite formation and the unresolved question of NAD(+) synthesis during inflammation and infection. Front Immunol. (2020) 11:31. doi: 10.3389/fimmu.2020.00031 32153556 PMC7047773

[50] Jamshed L Debnath A Jamshed S Wish JV Raine JC Tomy GT. An emerging cross-species marker for organismal health: tryptophan-kynurenine pathway. Int J Mol Sci. (2022) 23:6300–25. doi: 10.3390/ijms23116300 35682980 PMC9181223

[51] Chen Y Guillemin GJ. Kynurenine pathway metabolites in humans: disease and healthy States. Int J Tryptophan Res. (2009) 2:1–19. doi: 10.4137/IJTR.S2097 22084578 PMC3195227

[52] Sundaram G Lim CK Brew BJ Guillemin GJ. Kynurenine pathway modulation reverses the experimental autoimmune encephalomyelitis mouse disease progression. J Neuroinflamm. (2020) 17:176. doi: 10.1186/s12974-020-01844-y PMC7276083 32505212

[53] Akimov MG Dudina PV Fomina-Ageeva EV Gretskaya NM Bosaya AA Rudakova EV. Neuroprotective and antioxidant activity of arachidonoyl choline, its bis-quaternized analogues and other acylcholines. Dokl Biochem Biophys. (2020) 491:93–7. doi: 10.1134/S1607672920020027 32483760

[54] Akimov MG Kudryavtsev DS Kryukova EV Fomina-Ageeva EV Zakharov SS Gretskaya NM. Arachidonoylcholine and other unsaturated long-chain acylcholines are endogenous modulators of the acetylcholine signaling system. Biomolecules. (2020) 10:283–300. doi: 10.3390/biom10020283 32059521 PMC7072677

[55] Hwang JS Shin YJ. Role of choline in ocular diseases. Int J Mol Sci. (2021) 22:4733–45. doi: 10.3390/ijms22094733 33946979 PMC8124599

[56] Garcia M Mamedova LK Barton B Bradford BJ. Choline Regulates the Function of Bovine Immune Cells and Alters the mRNA Abundance of Enzymes and Receptors Involved in Its Metabolism *in vitro* . Front Immunol. (2018) 9:2448. doi: 10.3389/fimmu.2018.02448 30410489 PMC6211314

[57] Sanchez-Lopez E Zhong Z Stubelius A Sweeney SR Booshehri LM Antonucci L. Choline uptake and metabolism modulate macrophage IL-1beta and IL-18 production. Cell Metab. (2019) 29:1350–62.e7. doi: 10.1016/j.cmet.2019.03.011 30982734 PMC6675591

[58] Ma H Wang X Zheng X Wei H. Sphingomyelin is a prospective metabolic immune checkpoint for natural killer cells. Clin Transl Med. (2023) 13:e1395. doi: 10.1002/ctm2.1395 37649247 PMC10468581

[59] Lee M Lee SY Bae YS. Functional roles of sphingolipids in immunity and their implication in disease. Exp Mol Med. (2023) 55:1110–30. doi: 10.1038/s12276-023-01018-9 PMC10318102 37258585

[60] Lei Y Yang J Li H Zhong H Wan Q. Changes in glucose-lipid metabolism, insulin resistance, and inflammatory factors in patients with autoimmune thyroid disease. J Clin Lab Anal. (2019) 33:e22929. doi: 10.1002/jcla.22929 31350776 PMC6757119

[61] Nutt SL Hodgkin PD Tarlinton DM Corcoran LM. The generation of antibody-secreting plasma cells. Nat Rev Immunol. (2015) 15:160–71. doi: 10.1038/nri3795 25698678

[62] Chihara N Matsumoto R Yamamura T. Plasmablasts and neuroimmunological disorders. Immunol Med. (2019) 42:103–07. doi: 10.1080/25785826.2019.1659476 31464170

[63] Steinmetz TD Verstappen GM Suurmond J Kroese FGM. Targeting plasma cells in systemic autoimmune rheumatic diseases - Promises and pitfalls. Immunol Lett. (2023) 260:44–57. doi: 10.1016/j.imlet.2023.06.005 37315847

[64] Di Ciaula A Garruti G Lunardi Baccetto R Molina-Molina E Bonfrate L Wang DQ. Bile acid physiology. Ann Hepatol. (2017) 16:s4–s14. doi: 10.5604/01.3001.0010.5493 29080336

[65] Song X Sun X Oh SF Wu M Zhang Y Zheng W. Microbial bile acid metabolites modulate gut RORgamma(+) regulatory T cell homeostasis. Nature. (2020) 577:410–15. doi: 10.1038/s41586-019-1865-0 PMC7274525 31875848

[66] Xiao R Lei K Kuok H Deng W Zhuang Y Tang Y. Synthesis and identification of lithocholic acid 3-sulfate as RORgammat ligand to inhibit Th17 cell differentiation. J Leukoc Biol. (2022) 112:835–43. doi: 10.1002/JLB.1MA0122-513R 35188700

[67] Godlewska U Bulanda E Wypych TP. Bile acids in immunity: Bidirectional mediators between the host and the microbiota. Front Immunol. (2022) 13:949033. doi: 10.3389/fimmu.2022.949033 36052074 PMC9425027

[68] Tippalagama R Singhania A Dubelko P Lindestam Arlehamn CS Crinklaw A Pomaznoy M. HLA-DR marks recently divided antigen-specific effector CD4 T cells in active tuberculosis patients. J Immunol. (2021) 207:523–33. doi: 10.4049/jimmunol.2100011 PMC8516689 34193602

[69] Jacobson EM Huber A Tomer Y. The HLA gene complex in thyroid autoimmunity: from epidemiology to etiology. J Autoimmun. (2008) 30:58–62. doi: 10.1016/j.jaut.2007.11.010 18178059 PMC2244911

[70] Wang C Liu X Li Z Chai Y Jiang Y Wang Q. CD8(+)NKT-like cells regulate the immune response by killing antigen-bearing DCs. Sci Rep. (2015) 5:14124. doi: 10.1038/srep14124 26369936 PMC4569892

