# Supplementary material for: Genetically predicted metabolites mediate the causal associations between autoimmune thyroiditis and immune cells
Source: Front Endocrinol (Lausanne). 2024 Jul 9;15:1424957. doi: 10.3389/fendo.2024.1424957 (PMC11263034; doi:10.3389/fendo.2024.1424957)
Supplement: Supplementary file 3 [file Table_2.pdf]

STROBE-MR checklist of recommended items to address in reports of Mendelian randomization studies[12]

Item No.	Section	Checklist item	Section No.	Relevant text from manuscript
1	**TITLE and ABSTRACT**	Indicate Mendelian randomization (MR) as the study's design in the title and/or the abstract if that is a main purpose of the study	Title	Genetically predicted metabolites mediate the causal associations between autoimmune thyroiditis and immune cells
			Abstract	"We aimed to comprehensively investigate the causal relationship between 731 immune cell traits and autoimmune thyroiditis (AIT) and to identify and quantify the role of 1400 metabolic traits as potential mediators in between. Using summary-level data from genome-wide association studies (GWAS) we performed a two-sample bidirectional Mendelian randomization (MR) analysis of genetically predicted AIT and 731 immune cell traits. Furthermore, we used a two-step MR analysis to quantify the proportion of the total effects (that the immune cells exerted on the risk of AIT) mediated by potential metabolites. We identified 24 immune cell traits (with odds ratio (OR) ranging from 1.3166 6 to 0.6323) and 10 metabolic traits (with OR ranging from 1.7954 to 0.6158) to be causally associated with AIT, respectively. Five immune cell traits (including CD38 on IgD+ CD24-, CD28 on CD28+ CD45RA+ CD8br, HLA DR+ CD4+ AC, TD CD4+ %CD4+, and CD8 on EM CD8br) were found to be associated with the risk of AIT, which were partially mediated by metabolites (including glycolithocholate sulfate, 5alpha-androstan-3alpha,17beta-diol disulfate, arachidonoylcholine, X-15486, and kynurenine). The proportion of genetically predicted AIT mediated by the identified metabolites could range from 5.58% to 17.7%. In conclusion, our study identified causal associations between AIT and immune cells which were partially mediated by metabolites, thus providing guidance for future clinical and basic research."

	**INTRODUCTION**		1	
2	**Background**	Explain the scientific background and rationale for the reported study. What is the exposure? Is a potential causal relationship between exposure and outcome plausible? Justify why MR is a helpful method to address the study question	1	The contents of Introduction include general information of AIT, potential causal relationship between AIT and immune cells, potential causal relationship between immune cells and metabolites, potential causal relationship between AIT and metabolites, and that MR is a helpful method to address the study question.
3	**Objectives**	State specific objectives clearly, including pre-specified causal hypotheses (if any). State that MR is a method that, under specific assumptions, intends to estimate causal effects	1	"In the present study, we aimed to determine the specific immune cell signature that was causally associated with AIT and to assess the extent to which a specific metabolic trait could mediate the effect of the immune cell on AIT."
	**METHODS**		2	
4	**Study design and data sources**	Present key elements of the study design early in the article. Consider including a table listing sources of data for all phases of the study. For each data source contributing to the analysis, describe the following:	2.1-2.3	
	a)	Setting: Describe the study design and the underlying population, if possible. Describe the setting, locations, and relevant dates, including periods of recruitment, exposure, follow-up, and data collection, when available.	2.1	"Figure 1 shows a schematic summary of the analysis….This Study is conducted and reported following the Strengthening the Reporting of Observational Studies in Epidemiology Using Mendelian Randomization guidelines (STROBE-MR) (22)."
	b)	Participants: Give the eligibility criteria, and the sources and methods of selection of participants. Report the sample size, and whether any power or sample size calculations were carried out prior to the main analysis	2.2.1-2.2.3	GWAS data source for AIT and HT.<br>GWAS data source for 731 immune cell traits.<br>GWAS data source for 1400 metabolic traits.
	c)	Describe measurement, quality control and selection of genetic variants	2.3	"To estimate causal effects using genetic variation, three basic assumptions of IVs must be satisfied…where K is the number of genetic variants, N is the sample size. SNPs with an F statistic <10 should be excluded (28)."
	d)	For each exposure, outcome, and other relevant variables, describe methods of assessment and diagnostic criteria for diseases	2.2	"Autoimmune thyroiditis" including Hashimoto thyroiditis, hashitoxicosis (transient), lymphadenoid goiter, lymphocytic thyroiditis, struma lymphomatosa were defined as AIT cases

					(https://icd.who.int/browse10/2016/en#/E06.3).”
		e)	Provide details of ethics committee approval and participant informed consent, if relevant	2.2	“The datasets used in our analysis were publicly available and were approved by the institutional review committee in the respective studies. Therefore, no further sanctions were needed.”
5	**Assumptions**		Explicitly state the three core IV assumptions for the main analysis (relevance, independence and exclusion restriction) as well assumptions for any additional or sensitivity analysis	2.1	“We ensured a robust association existing between genetic variants and the exposure (assumption 1); we ascertained that these genetic variants are not associated with potential confounding factors (assumption 2); and we confirmed that the impact of genetic variants on the outcome is mediated exclusively through the exposure, without the influence of alternative biological pathways (assumption 3).”
6	**Statistical methods: main analysis**		Describe statistical methods and statistics used	2.1-2.4	
		a)	Describe how quantitative variables were handled in the analyses (i.e., scale, units, model)	2.1	Quantitative variables in the analyses can be referred from each GWAS data sources for AIT, HT, immune cell traits, and metabolic traits.
		b)	Describe how genetic variants were handled in the analyses and, if applicable, how their weights were selected	2.3	“To estimate causal effects using genetic variation, three basic assumptions of IVs must be satisfied…where K is the number of genetic variants, N is the sample size. SNPs with an F statistic <10 should be excluded (28).”
		c)	Describe the MR estimator (e.g. two-stage least squares, Wald ratio) and related statistics. Detail the included covariates and, in case of two-sample MR, whether the same covariate set was used for adjustment in the two samples	2.4.1	“Inverse variance weighting (IVW) uses meta-analysis to combine the Wald ratios of causal effects for each single nucleotide polymorphism (SNP) (29)…A significance threshold of P < 0.05 was applied (or otherwise indicated).”
		d)	Explain how missing data were addressed	2.4.1	“Missing values were not inferred.”
		e)	If applicable, indicate how multiple testing was addressed	2.4.1	“Then, MR-Egger (30), weighted-median (31), simple mode, and weighted mode methods (32) were used as a complement to IVW…”
7	**Assessment of**		Describe any methods or prior knowledge used to assess the assumptions or	2.4.1	“Inverse variance weighting (IVW) uses meta-

	**assumptions**	justify their validity		analysis to combine the Wald ratios of causal effects for each single nucleotide polymorphism (SNP) (29)…A significance threshold of $P < 0.05$ was applied (or otherwise indicated).”
8	**Sensitivity analyses and additional analyses**	Describe any sensitivity analyses or additional analyses performed (e.g. comparison of effect estimates from different approaches, independent replication, bias analytic techniques, validation of instruments, simulations)	2.5	“Heterogeneity was assessed using Cochrane's Q test calculated in the IVW methods while potential pleiotropy was evaluated and corrected using the MR-Egger intercept test. Cochran's Q test is employed as a method to evaluate heterogeneity among different IVs in a study (37)…”
9	**Software and pre-registration**		2.4-2.5	
	a)	Name statistical software and package(s), including version and settings used	2.4-2.5	“All the analyses were done by R package named “TwoSampleMR” within the environment of R 4.3.2 (available at https://cran.r-project.org/bin/windows/base/).”<br>“Furthermore, a powerful method, the MR pleiotropy residual sum and outlier (MR-PRESSO) method was utilized to exclude possible horizontal pleiotropic outliers that could substantially affect the estimation results in the MR-PRESSO package (38).”
	b)	State whether the study protocol and details were pre-registered (as well as when and where)	-	Not applicable.
	**RESULTS**		3	
10	**Descriptive data**			
	a)	Report the numbers of individuals at each stage of included studies and reasons for exclusion. Consider use of a flow diagram	2.2	Details could be found in each original study included in the public data (see data sources).
	b)	Report summary statistics for phenotypic exposure(s), outcome(s), and other relevant variables (e.g. means, SDs, proportions)	2.2	Details could be found in each original study included in the public data (see data sources).
	c)	If the data sources include meta-analyses of previous studies, provide the assessments of heterogeneity across these studies	3.1-3.4	Analysis results for heterogeneity were all included in Supplementary Tables S4, S7, S11, S14, S17, S21, S25, and S28.

		d)	For two-sampleMR:<br>i. Provide justification of the similarity of the genetic variant-exposure associations between the exposure and outcome samples<br>ii. Provide information on the number of individuals who overlap between the exposure and outcome studies	3.1-3.4	Characteristics of significant SNPs with genome-wide associations for exposure and outcome were all included in Supplementary Tables S2, S9, and S16, S20, and S24.
11	**Main results**			3	
		a)	Report the associations between genetic variant and exposure, and between genetic variant and outcome, preferably on an interpretable scale	3.1<br>3.2<br><br>3.3<br>3.4	Associations between immune cells and AIT.<br>Associations between immune cells and HT (MR validation).<br>Associations between metabolites and AIT.<br>Associations between immune cells and metabolites.
		b)	Report MR estimates of the relationship between exposure and outcome, and the measures of uncertainty from the MR analysis, on an interpretable scale, such as odds ratio or relative risk per SD difference	3.1<br><br><br>3.2<br><br>3.3<br><br>3.4	Information including OR, 95% CI, and p values for immune cells associated with AIT; the data were all included in Figure 2-3, Supplementary Figure S1.<br>Information including OR, 95% CI, and p values for HT associated with immune cells; the data were all included in Supplementary Figure S2.<br>Information including OR, 95% CI, and p values for metabolites associated with AIT/HT; the data were all included in Figure 4, Supplementary Figure S3-4.<br>Information including OR, 95% CI, and p values for immune cells associated with metabolites; the data were all included in Figure 5, Supplementary Figure S6.
		c)	If relevant, consider translating estimates of relative risk into absolute risk for a meaningful time period		Not applicable.
		d)	Consider plots to visualize results (e.g. forest plot, scatter plot of associations between genetic variants and outcome versus between genetic variants and exposure)	3.1-3.5	Forest plots: Figures 2-6, and Supplementary Figures S1-S4, and S6-S7.<br>Scatter plots: Supplementary Figures S8-S10;<br>Funnel plots: Supplementary Figures S11-S13;<br>“Leave-one-out sensitivity” plots: Supplementary

					Figures S14-S16.
12	**Assessment of assumptions**			3	
		a)	Report the assessment of the validity of the assumptions	3.1-3.4	Assessments of the validity of the assumptions employed five methods, and were all included in Figure 2-6; Supplementary Tables S1, S5, S6, S8, S12, S13, S15, S19, S23, and S27; Supplementary Figures S1-S4, and S6-S7..
		b)	Report any additional statistics (e.g., assessments of heterogeneity across genetic variants, such as I^2, Q statistic or E-value)	3.1-3.5	Assessments for heterogeneity were all included in Supplementary Tables S4, S7, S11, S14, S17, S21, S25, and S28.
13	**Sensitivity analyses and additional analyses**			3	
		a)	Report any sensitivity analyses to assess the robustness of the main results to violations of the assumptions	3.1-3.5	Sensitivity analyses for horizontal pleiotropy and heterogeneity were all included in Supplementary Tables S3, S4, S7, S10, S11, S14, S17, S18, S21, S22, S25, S26, S28, and S29.
		b)	Report results from other sensitivity analyses or additional analyses	3.5	Leave-one-out analyses were all included in Supplementary Figures S14-S16.
		c)	Report any assessment of direction of causal relationship (e.g., bidirectional MR)	3.1-3.2	Results of reverse causal associations of AIT or HT with immune cells were all included in Supplementary Tables S5-6, S12-13, and Figure 3.
		d)	When relevant, report and compare with estimates from non-MR analyses		Not applicable.
		e)	Consider additional plots to visualize results (e.g., leave-one-out analyses)	3.5	The results were all included in Supplementary Figures S14-S16.
	**DISCUSSION**			4	
14	**Key results**		Summarize key results with reference to study objectives	4	"We identified 27 immune cells and 10 metabolites to be causally associated with AIT (at least in the IVM method), among which 5 immune cells…The results suggest that metabolites were able to, at least partially, mediate the causal

				relationship between immune cells and AIT.”
15	**Limitations**	Discuss limitations of the study, taking into account the validity of the IV assumptions, other sources of potential bias, and imprecision. Discuss both direction and magnitude of any potential bias and any efforts to address them	4	“Nevertheless, there were several limitations in our study...”
16	**Interpretation**		4	
	a)	Meaning: Give a cautious overall interpretation of results in the context of their limitations and in comparison with other studies	4	“The results suggest that metabolites were able to, at least partially, mediate the causal relationship between immune cells and AIT.”
	b)	Mechanism: Discuss underlying biological mechanisms that could drive a potential causal relationship between the investigated exposure and the outcome, and whether the gene-environment equivalence assumption is reasonable. Use causal language carefully, clarifying that IV estimates may provide causal effects only under certain assumptions	4	The underlying biological mechanisms that could drive a potential causal relationship between immune cells and metabolite, metabolite and AIT, immune cells and AIT were thoroughly discussed in the section.
	c)	Clinical relevance: Discuss whether the results have clinical or public policy relevance, and to what extent they inform effect sizes of possible interventions	4	“Hopefully, our study provides new insights into the integration of immune cells and metabolites for further exploration of the biological mechanisms of AIT, and some guidance for potential therapeutic strategies for AIT.”
17	**Generalizability**	Discuss the generalizability of the study results (a) to other populations, (b) across other exposure periods/timings, and (c) across other levels of exposure	4	Study results were deemed generalizable at European level, and discussed as such: “First, our analysis was performed using the European population, which limits its prevalence.”
	**OTHER INFORMATION**		5-10	
18	**Funding**	Describe sources of funding and the role of funders in the present study and, if applicable, sources of funding for the databases and original study or studies on which the present study is based	7	“This work was supported by the National Natural Science Foundation of China (#81900712) and the Project of National Clinical Research Base of Traditional Chinese Medicine in Jiangsu Province, China (JD2023SZX12).”
19	**Data and data sharing**	Provide the data used to perform all analyses or report where and how the data can be accessed, and reference these sources in the article. Provide the statistical code needed to reproduce the results in the article, or report whether the code is publicly accessible and if so, where	5	“All datasets generated during the current study are included in the article/supplementary materials.”
20	**Conflicts of**	All authors should declare all potential conflicts of interest	6	“The authors declare that the research was conducted in the absence of any commercial or

**Interest**		financial relationships that could be construed as a potential conflict of interest."

This checklist is copyrighted by the Equator Network under the Creative Commons Attribution 3.0 Unported (CC BY 3.0) license.

1. Skrivankova VW, Richmond RC, Woolf BAR, Yarmolinsky J, Davies NM, Swanson SA, et al. Strengthening the Reporting of Observational Studies in Epidemiology using Mendelian Randomization (STROBE-MR) Statement. JAMA. 2021;under review.

2. Skrivankova VW, Richmond RC, Woolf BAR, Davies NM, Swanson SA, VanderWeele TJ, et al. Strengthening the Reporting of Observational Studies in Epidemiology using Mendelian Randomisation (STROBE-MR): Explanation and Elaboration. BMJ. 2021;375:n2233.
